# Correlation of PET-CT nodal SUVmax with p16 positivity in oropharyngeal squamous cell carcinoma

**DOI:** 10.1186/s40463-015-0091-5

**Published:** 2015-09-15

**Authors:** Jessica Clark, Caroline C. Jeffery, Han Zhang, Tim Cooper, Daniel A. O’Connell, Jeffrey Harris, Hadi Seikaly, Vincent L. Biron

**Affiliations:** Faculty of Medicine and Dentistry, Department of Surgery, Division of Otolaryngology-Head and Neck Surgery, University of Alberta, Edmonton, Alberta Canada; University of Alberta, Otolaryngology-Head and Neck Surgery, 1E4, Walter Mackenzie Centre, University of Alberta Hospital, 8440-112St, Edmonton, Alberta T6G 2B7 Canada

**Keywords:** Oropharyngeal cancer, Positron emission tomography, Standard uptake value, p16, Human papillomavirus

## Abstract

**Background:**

The incidence of human papillomavirus (HPV)-related oropharyngeal squamous cell carcinoma (OPSCC) has been rising in recent years. Given the clinical impact of HPV/p16 positivity in OPSCC, identifying surrogate markers of this disease early in the diagnostic work-up of these patients could improve patient care.

**Methods:**

Demographic, pathologic, staging and PET-CT data from patients diagnosed with OPSCC from 2009–2014 were obtained from a prospectively collected provincial cancer registry. Tumor HPV/p16 status was correlated to the maximum standard uptake value (SUVmax) of the primary tumor and cervical nodes. Comparisons of means and multinomial regression models were used to determine associations between p16 status and SUVmax. A diagnostic odds ratio was calculated using a cut off value for predicting HPV/p16 positivity based on nodal SUVmax.

**Results:**

PET-CT and HPV/p16 data was obtained for 65 patients treated surgically for OPSCC. Significantly higher nodal SUVmax was associated with HPV/p16 positive nodes (SUVmax 10.8 vs 7.9). No significant differences were seen between HPV/p16 positive vs negative primary tumor SUVmax (10.3 vs 13.7). In combination with other clinical parameters, higher nodal SUVmax was highly correlated with HPV/p16 positivity.

**Conclusion:**

Elevated nodal SUVmax is a significant predictor of HPV/p16 positive disease.

## Introduction

Oropharyngeal squamous cell carcinoma (OPSCC) is an aggressive malignancy with a rising incidence worldwide [1–3]. Oncogenic human papillomavirus (HPV) infection, tobacco and alcohol are well established etiological factors for OPSCC [4, 5]. A large body of evidence from recent years has demonstrated that HPV positive and negative OPSCC are distinct from clinical, pathological and molecular perspectives [4, 6–12]. Most importantly; patients with HPV-related OPSCC, generally pathologically identified by p16 positivity, have favorable survival outcomes following both surgical and non-surgical treatment approaches [4, 5, 13]. As recommended by recent head and neck cancer treatment guidelines, it is important to establish a pathologic diagnosis of HPV/p16 OPSCC for purposes of prognostication [14]. Despite well-characterized clinical and histopathological differences, the treatment algorithm for HPV/p16 positive and negative OPSCC remains unchanged.

Positron emission tomography-computed tomography (PET-CT) is an important tool for the diagnosis and surveillance of OPSCC [12, 15, 16]. PET-CT utilizes glucose metabolism to identify tumor metabolic activity, which can accurately identify locoregional disease better than CT alone, particularly in smaller nodes [17, 18]. Rates of cervical lymph node metastases in HPV positive OPSCC have been reported to be 60-76 % at initial presentation [19]. Sensitivity and specificity of detecting cervical node metastases with PET-CT is high, ranging from 84-92 % and 95-99 %, respectively [18]. HPV positive nodal metastases often present as a cystic or necrotic neck mass which may be less metabolically active on PET [20]. Additionally, inflammatory nodes can present as FDG avid lymphadenopathy and could represent false positives on PET imaging. Combining PET with CT allows for precise anatomic localization and further characterization of these potential metastatic lymph nodes.

The clinical role of PET-CT to localize primary tumors and metastatic disease has been well-established; but researchers are continuing to try to identify specific imaging biomarkers that may help diagnose, provide information about prognosis and guide treatments for patients with OPSCC. Maximum standard uptake value (SUVmax) is a quantitative measure of the highest FDG uptake within the region of interest. It has been hypothesized by others that a high SUVmax would be associated with a poor outcome, reflecting the increased proliferation of the tumor cells [21]. However HPV positive tumors, known to have favorable outcomes, are thought to have higher metabolic activity with increased proliferation and a predilection for early metastasis [22, 23]. Recent data from Kendi *et al.* [24] support the hypothesis that higher SUVmax is associated with HPV/p16 positivity. There is therefore conflicting evidence regarding the association between SUVmax and HPV/p16 positivity in OPSCC [21, 24–26]. To this end, we aimed to determine the correlation between nodal SUVmax and p16 status of OPSCC tumors using a cohort of patients treated with primary surgery.

## Methods

The University of Alberta Research Ethics Board granted ethics approval for the study (Pro00016426).

### Patients

**Fig. 1:**
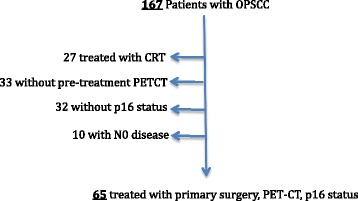
Inclusion and exclusion criteria. OPSCC – oropharyngeal squamous cell carcinoma, CRT – chemoradiation, PET-CT – positron emission tomography – computed tomography, N0 – no nodal disease. Patients who were treated with initial surgical resection and neck dissection +/− radiation/chemoradiation were considered to have primary surgical treatment

Patients were identified using a prospectively collected database (Alberta Cancer Registry [27] (ACR)) of patients treated for OPSCC from January 2009 – December 2014 at a tertiary cancer treatment facility in Alberta, Canada. All adult patients treated with a primary surgical approach with a pre-treatment PET-CT and p16 status of the OPSCC were included within the cohort (Fig. 1). Patients were categorized as smokers using a previously defined cutoff of ≥ 10 pack years of tobacco use [4]. Patients were excluded if they had received previous treatment for head and neck cancer or found to have an N0 neck on surgical pathology. Initial demographic information was obtained from the ACR and verified by retrospective review of patients’ charts. HPV-status was obtained by immunohistochemistry staining for p16, a surrogate marker of oncogenic HPV, as previously reported using established standards [11, 13, 28, 29].

### PET-CT image analysis

PET-CT protocols were obtained using two scanners, both Gemini TF 16-slice PET-CT scanner systems (Phillips Healthcare, Andover, MA). All patients were scanned with a consistent head and neck protocol. Following a fasting period of at least 4 h and serum baseline glucose measurements, patients were injected with 5.18 MBq/kg of 18-FDG tracer. A minimum of 60 min was waited following tracer injection and PET scanning. Two separate acquisitions were used to scan patients from skull vertex to mid thighs. Following the PET scan, patients were injected with intravenous contrast and a helical CT was performed to scan patients from skull vertex to mid thighs.

Using OASIS software (Segami Corporation, Columbus, Ohio), PET-CT images were reviewed by authors JC, CJ and TC. Reviewers were blinded to p16 status at the time of imaging review. Coronal, sagittal and transverse images were examined to identify SUVmax of the primary tumor and cervical nodal metastases. A spherical volumetric area of interest was focused upon individual lesions in the head and neck to identify metabolic parameters of the lesions. Final radiological and nuclear medicine reports were reviewed for additional correlation of findings. Only nodal and primary malignancies with FDG uptake were included within the analysis. The SUVmax for the primary as well as the nodal SUVmax were obtained from the OASIS software. In patients with multiple FDG avid cervical lymph nodes, the highest SUVmax was recorded, consistent with previous PET-CT protocols [25].

### Statistical analyses

SPSS version 23.0 was used for statistical analyses (SPSS Inc., Chicago, IL, USA). Regression analyses of factors and covariates were performed to include age, gender, TNM staging, tumor subsite, treatment, smoking status, p16 positivity and SUVmax. ANOVA, Mann Whittney *U* test, and the Kruskal-Wallis test were used to calculate differences between groups where appropriate. Statistical significance was defined as p < 0.05.

## Results

A prospectively collected database identified 167 patients who were treated at the University of Alberta for OPSCC between 2009 and 2014. Of the patients treated with primary surgery, sixty-five had adequate pre-treatment PET-CT and p16 status available (Fig. 1).

Patients with p16 negative disease were more likely to have a smoking history (>10 pack years) (Table 1). No statistically significant differences were seen between p16 positive and negative patients in terms of age at presentation, gender, T stage and N stage.

**Table 1:** Demographics and staging of oropharyngeal malignancies

Characteristic	P16+	P16-	*P* value
Age (mean, SD)	57.6, 7.6	59.5, 10.6	0.39
Gender (% male)	88.2	76.9	0.06
Smoking	54.9	84.6	< 0.001
Tumor stage			
T1	15.3	25.4	0.09
T2	23.1	41.1	
T3	15.3	15.7	
T4	46.2	17.6	
Nodal stage			
N1	38.5	13.7	0.41
N2	53.8	80.4	
N3	7.7	5.9	

*SD* standard deviation

**Fig. 2:**
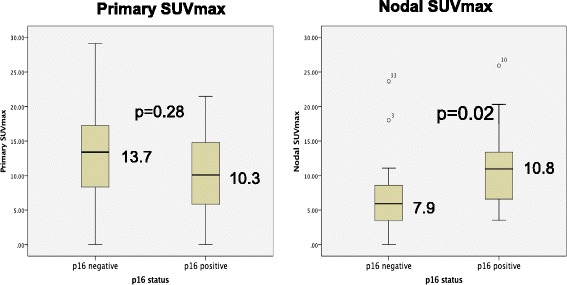
Comparison of means for p16 positive and negative disease for primary and nodal maximum standard uptake value (SUVmax)

The mean SUV max of the primary tumor for p16 negative disease (13.7) was higher than that for p16 positive disease (10.3) but there was no significant difference when these were assessed with a Mann Whitney U comparison of means (p = 0.28). The average nodal SUVmax for p16 positive disease (10.8) was significantly higher (p = 0.02) than that for p16 negative disease (7.9) (Fig. 2).

Using the dependent variable, p16 status, a multinomial logistic regression analysis was performed (Table 2). The only variable that was found to be significant in predicting p16 status was nodal SUVmax. Higher nodal SUVmax was associated with p16 positive disease. Primary tumor SUVmax was not a significant predictor.

**Table 2:** Multinomial analysis with p16 status as the dependent variable

Covariate	Standardized coefficient	*P* value
Gender	−0.046	0.820
T stage	0.113	0.594
N stage	0.027	0.895
Smoking	−0.075	0.712
ECOG	−0.270	0.158
ECS	0.023	0.918
Primary SUV max	−0.292	0.183
Nodal SUVmax	0.480	0.041

*ECOG* Eastern Cooperative Oncology Group performance status, *ECS* extra-capsular spread, *SUVmax* maximum standard uptake value

Using the mean SUVmax of p16 positive nodes as a significant predictor of p16 positive disease, we categorized patients according to this cutoff value (mean SUVmax p16+). Patients with nodal SUVmax ≥ mean SUVmax p16+ had a positive predictive value of p16 positive disease of 92.3 %. The diagnostic odds ratio (DOR) of the mean SUVmax p16+ was 4.9 (95 % CI, 1.0-24.3)

## Discussion

The emergence of PET-CT as a diagnostic tool for head and neck malignancies has allowed physicians to identify primary tumors and locoregional metastases early in the disease course. Many feel the role of PET-CT can be expanded to help characterize some of these lesions to more accurately diagnose and target therapy for different types of cancers. This study has shown a significant association between higher nodal SUVmax and p16 positive disease status. In addition, in patients who had a nodal SUVmax of greater than 10.8 (mean SUVmax p16+), we could reliably predict p16 positive disease (DOR = 4.9). A biological basis for these findings could represent the higher cell division and oxygenation of p16 positive tumors or the immune response of HPV infected nodes, which could FDG activity [24].

Two similar studies examining the association of PET-CT and HPV status have found conflicting results [24, 25]. Tahari and colleagues had a large cohort (N = 123) and retrospectively examined the relationship between PET-CT imaging markers and HPV status [25]. They found higher PET values in HPV positive nodal metastasis but this difference was not statistically significant. Kendi and colleagues [24] examined the relationship of PET-CT imaging markers and p16 status in patients with oral cavity and OPSCC . Consistent with results from our study, they found a statistically significantly higher nodal SUVmax, but not primary tumor SUVmax, in patients with HPV positive disease as compared to those with HPV negative cancer. Although their study had a relatively small sample size (N = 22), they also identified a nodal SUVmax cut off value, which was predictive of HPV positivity (SUVmax >7.66). Our study supports a significant association between p16 status and nodal SUVmax. With this data, clinicians may be able to more accurately predict HPV status based on imaging in combination with other clinical parameters.

HPV positive tumors are being found in a new population – younger males without environmental risk factors such as tobacco and alcohol. Clinicians; however, may be slow to screen these individuals for malignancies, as they do not have the traditional exposures that have been historically associated with head and neck cancer. As a result, the average time for diagnosis of OPSCC is greater than 3 months from initial presentation at a general practioner’s office [30]. Given the prognostic importance of p16 positivity in OPSCC, diagnostic information that can assist in triaging patients according to p16 status is valuable to both patients and physicians.

PET-CT also plays a role in identifying primary tumors in cases of cancer of unknown primary (CUP). Multiple studies have found HPV positive metastatic neck nodes are more likely to be associated with an oropharyngeal primary than other head and neck malignancies [31, 32]. Nodal SUVmax, identified on pre-treatment PET-CT could give additional information about the underlying malignancy for CUP. Having a high suspicion for p16 positive OPSCC based on imaging could help direct a more aggressive search for a CUP. Recent reports using transoral resection of base of tongue and tonsils in p16 positive CUPs have been >90 % effective in identifying the primary site [33, 34].

Our findings could have a particularly useful clinical implication in the diagnosis of OPSCC patients when surgical biopsy under general anesthesia may portend a high risk of morbidity. If imaging with PET-CT could help identify p16 status, clinicians may be able to tailor chemoradiation therapy in this population. It is thought the improved prognosis of HPV positive OPSCC relates in part to the increased radiosensitivity of the tumor. Currently HPV positive and negative tumors are treated equivocally. Given the variable pathologic and molecular behavior of HPV positive and negative tumors, targeted treatments may soon be developed that could allow for individualized therapies that may reduce overall radiation-related side effects and improve post-treatment quality of life.

This study has a number of limitations. Although initial data were collected prospectively, it remains a retrospective analysis in a single institution, which could limit the generalizability of the results. As shown in Fig. 1, several patients were excluded from the analysis due to lack of PET-CT or p16 status, which could bias our results. PET-CT protocols may vary between cancer treatment facilities, which may limit the extrapolation of nodal SUVmax cutoff values described herein. Finally, a relatively small patient cohort was used in this analysis. These limitations could be improved with further studies using prospective data from multiple institutions.

## Conclusions

There is a significant association between p16 positivity and increased nodal SUVmax in OPSCC. Further larger scale studies involving other institutions would be recommended to examine the clinical utility of this association.
